# A randomized controlled trial of HighScope’s teacher professional learning on preschoolers’ executive function skills

**DOI:** 10.1016/j.ecresq.2026.03.012

**Published:** 2026-03-20

**Authors:** Sammy F. Ahmed, Nicholas E. Waters, Lori E. Skibbe

**Affiliations:** a University of Rhode Island, 2 Lower College Rd, Kingston RI 02881, USA; b Indiana University, 1101 E 10th St., Bloomington, IN 47405, USA; c Michigan State University, 552 W Circle Dr, East Lansing, MI 48824, USA

**Keywords:** HighScope, Teacher professional development, Teacher professional learning, Coaching, Executive function, Preschool

## Abstract

Teacher professional learning has been shown to promote children’s language, literacy, and social-emotional functioning - however, less is known about its impact on children’s executive function. In the present study, we employed a randomized controlled trial to understand whether HighScope’s teacher professional learning workshops and coaching can promote children’s executive function development during preschool. Teachers in the treatment group (*n* = 22) participated in 5 training workshops and received coaching on curricular implementation strategies, whereas teachers in the control group (*n* = 20) did not receive any training or coaching. Results from a sample of 317 children enrolled in publicly funded preschools (*M*_age_=49.8 months; 55% Female; 38% White, 36.8% Black, 14.4% multiracial, 4.4% Asian/Pacific Islander, 6.4% “other;” 6% Latine) revealed a positive effect (*ß*=0.140, *p*=0.019) of teacher professional learning on growth in children’s executive function from the fall to spring of preschool. Despite being statistically significant, the relatively small effect size might limit the practical impact on children’s executive function skills during preschool. Overall, the HighScope professional learning package shows promise for supporting children’s executive function development, yet the modest effect size underscores the need to better understand the ways that supporting teachers can promote children’s executive function development in early classroom settings.

As national investments in early childhood education and care continue to grow in the United States, it is becoming increasingly important to understand the effectiveness of teacher professional learning (PL) for improving children’s outcomes. PL typically consists of interactive learning experiences for early childhood educators that are designed to support the acquisition of knowledge and skills, as well as their application in classroom settings (Bergmark, 2023; Labone & Long, 2016). An important feature of PL programs involves coaching, whereby an expert observes teachers’ instruction, provides pedagogical feedback, and supports teachers’ implementation of curricula (Darling-Hammond et al., 2017; Neuman & Cunningham, 2009). PL workshops and coaching have been shown to improve instruction, classroom quality, and teacher-child interactions (Early et al., 2017; Pianta et al., 2008). For example, Domitrovich and colleagues (2009) found that Head Start teachers who participated in workshops and received in-class coaching interacted with students more frequently, and in more complex ways, employed more preventative classroom management strategies, and fostered more positive climates in their classrooms.

Teacher PL has also been shown to promote child outcomes during preschool. A recent meta-analysis of over 30,000 children found positive effects of workshops and coaching on children’s language and literacy skills, social-emotional development, and other important school readiness indicators (Brunsek et al., 2020). However, the extent to which teacher PL can reliably improve preschooler’s executive function remains unclear. Given the role of executive function in children’s learning and development (see Zelazo et al., 2016, for review), it is important to understand how PL can promote the development of these skills. Therefore, in the present study, we employed a randomized controlled trial to examine whether HighScope’s teacher PL package can promote children’s executive function development during preschool.

## 1. Teacher training workshops and coaching on children’s executive function skills

Executive function (EF) refers to a set of cognitive skills that underlie purposeful, goal-oriented behavior and includes working memory, inhibitory control, and cognitive flexibility (Zelazo et al., 2016). These foundational skills allow individuals to exert control over their behavior and play an essential role in everyday functions such as learning, reasoning, and problem-solving (Diamond, 2013). Although EF development is protracted into adulthood, interest in its early development has steadily mounted over the past two decades (e.g., Ahmed et al., 2022). This is largely due to EF’s accelerated development during the early childhood period, as well as its role in supporting children’s learning and development across the lifespan (Ahmed et al., 2019, 2021; Waters et al., 2021). As a result, there have been growing efforts to develop teacher PL programs designed to support the development of children’s EF skills during preschool.

Despite these efforts, evidence for the efficacy of teacher PL programs for promoting children’s EF skills during preschool has been mixed (see Muir et al., 2023, for meta-analysis). For example, Raver et al. (2011) found positive effects of the Chicago School Readiness Project (CSRP), which included teacher training workshops and coaching, on children’s EF skills at the end of preschool. CSRP provided teachers with training on classroom management strategies with a specific focus on managing children’s disruptive behavior. Additionally, teachers received feedback during in-class coaching sessions on the strategies learned in the training sessions and were provided with a mental health counselor to reduce teacher stress and burnout. In a follow-up study, McCoy et al. (2019) reported that CSRP’s effects on early academic skills mediated the intervention’s long-term impacts on EF skills during adolescence, providing additional evidence of CSRP’s efficacy. Conversely, Morris and colleagues (2014) reported null effects of the Head Start Cares program on children’s EF development during preschool. Head Start Cares paired different evidence-based curricula (i. e., Preschool PATHS, Incredible Years, and Tools of the Mind–Play) with teacher training and weekly coaching sessions. Although the intervention led to significant changes in teachers’ classroom practices and, in some cases, promoted children’s social and emotional skills, none of the programs had significant impacts on children’s EF at the end of preschool. Similarly, Ansari & Pianta (2018) reported non-significant effects of a digital coaching intervention on children’s EF skills. Specifically, coaches in the MyTeachingPartner (MTP) program provided teachers with feedback and led reflection and planning sessions with teachers to improve their interactions with students. Despite reporting improvements in teachers’ instructional support, there were no effects of MTP on children’s EF skills at the end of preschool (Ansari & Pianta, 2018).

Many of these interventions, however, have focused on global classroom management practices and teacher-student interactions meant to promote a wide range of child behaviors and outcomes, including language, literacy, math, social-emotional development, and externalizing problem behaviors. It may be the case that these types of school readiness interventions are too broad to reliably improve children’s EF skills, which could explain the mixed findings in the literature. It is also possible that the focus on several developmental domains makes it difficult for teachers to effectively implement these strategies into their daily classroom activities, which may reduce full implementation and contribute to teacher burnout (Nesbitt & Farran, 2021). Moreover, it has been hypothesized that PL workshops and coaching with an explicit focus on promoting children’s EF and self-regulation may be an effective approach (Mattera et al., 2021; Nesbitt & Farran, 2021; Weiland & Yoshikawa, 2013). A recent study by Muir and colleagues (2024) provides quasi-experimental evidence that EF-focused PL might, in fact, be an effective lever for improving preschoolers’ EF skills. They found that a teacher PL program that introduced teachers to the theoretical relevance of EF and self-regulation for children’s learning and development, as well as ways to foster EF skills in a classroom setting, produced positive intervention effects (Muir et al., 2024). This study suggests that programs that use EF-focused PL as a mechanism of change might be effective in promoting preschoolers EF development.

## 2. HighScope’s teacher PL model

HighScope’s PL model involves workshops and coaching by an experienced educator to support the implementation of HighScope’s curricular components [*Plan-Do-Review* (PDR) and *Conflict Resolution* (CR)] using instructional strategies learned during the training workshops. PDR and CR include an explicit focus on supporting EF by giving children repeated opportunities to practice core (i.e., working memory, cognitive flexibility, inhibition, and attention) and higher-level (i.e., planning, problem solving, and meta-cognition) EF skills, as well as self-regulation, by encouraging children to reason, negotiate, and develop language as they explain conflicts and agree on solutions. Specifically, in PDR, children plan their “work time” activities, execute their plan, and review their actions afterward. The process of planning, implementing, and recalling is aligned with EF skills (e.g., Moos & Ringdal, 2012). These strategies can help children develop attention (completing a task even when it is difficult), impulse control (inhibiting running to a new activity without thinking about the consequences of abandoning current work), working memory (recalling the original plan and reasons for changing plans), and behavioral and cognitive flexibility skills (changing plans to include new play partners). In CR, on the other hand, teachers serve as a neutral, third-party mediator as they work with children through six steps to discover a solution to a particular problem as the conflict occurs. The ability to propose a solution, agree on one together, and settle conflicts with peers is associated with regulating behavior and controlling impulses (Zelazo et al., 2016).

### 2.1. Teacher Training

Aligned with recent research on effective teacher trainings strategies (e.g., Muir et al., 2024), HighScope’s training model focuses on providing teachers with a theoretical understanding of the relevance of EF and self-regulation for children’s development, as well as specific strategies to promote these skills in a classroom setting. Training activities include strategies for implementing the PDR and CR curricular components and involve reflection and application activities with teachers. The ‘how’ and ‘why’ of the strategies are explained using extant literature on how children develop EF and self-regulation. Across training sessions, teachers experience parallels between how adults and children learn, while building in the curricular implementation strategies. The training structure does this by including a variety of learning experiences, from whole group discussions and guided practices to small group work and individual reflections. The active learning experiences in each training session were designed to help teachers make connections between the PDR and CR curricular components, the implementation strategies, and their classroom practices as they relate to how children develop EF and self-regulation skills.

### 2.2. Teacher Coaching

Coaches were trained to use a customized version of cognitive coaching as a framework when working with teachers, which is a relationship-based approach that builds on teachers’ strengths through conversations structured around planning, reflection, and problem-solving (Ellison & Hayes, 2009). This approach has been successfully used with school personnel to improve their professional competency (Rogers et al., 2017) and teachers report that it helps them become aware of their own practices, gain new knowledge, and try new approaches (Gyllensten et al., 2010). Coaches work in partnership with teachers to develop coaching plans based on observations of teacher strengths during critical times of the daily routine, and is focused on scaffolding during PDR (e.g., planning time, work time, and recall time) and teacher-student interactions during CR (e.g., encouraging children to reason, problem solve, and negotiate). Additionally, coaches build strong, trusting relationships with teaching teams/teachers by focusing on strengths in teachers’ practices, consulting with teachers in developing goals and plans, listening to and responding to concerns expressed by teachers, engaging teachers in brainstorming solutions to issues that occur, and exhibiting respect for teachers as experts in their own classrooms and communities. Importantly, coaches help teachers make connections between the theoretical underpinnings of the PDR and CR curricular components and specific classroom practices that promote children’s EF and self-regulation.

## 3. Current study

**Fig. 1.**
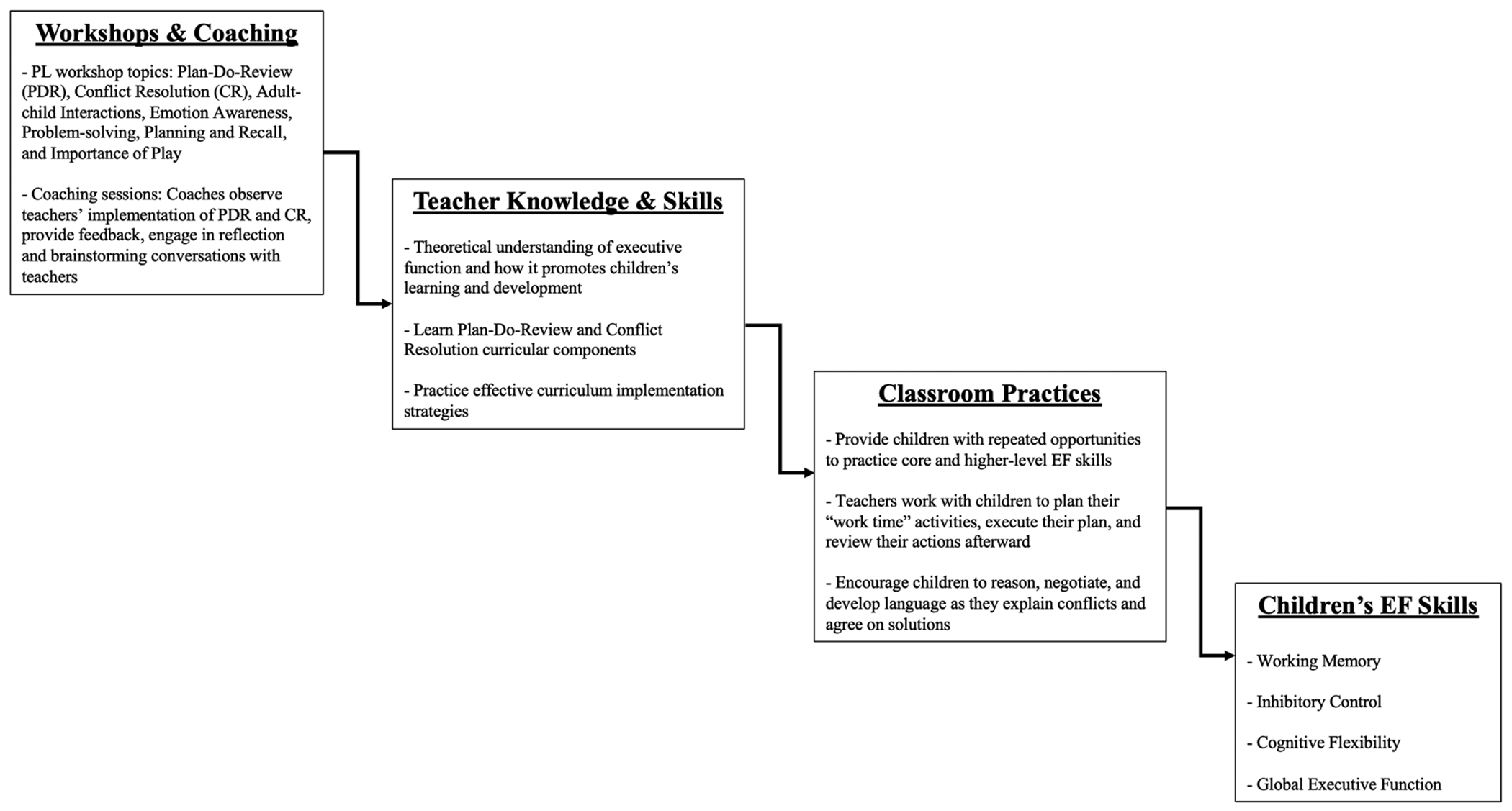
Theory of change from teacher pl workshops and coaching to children’s EF skills.

The primary research question motivating the current study is: What is the impact of HighScope’s teacher PL workshops and coaching on preschoolers’ EF development? As such, we employed a randomized controlled trial, using a pre- and post-test design. All teachers in the current study used HighScope’s preschool curriculum; however, only those in the treatment group participated in training workshops and received coaching on curriculum implementation. This design allowed us to isolate the impact of PL on children’s EF development across preschool. Based on previous research (e.g., Muir et al., 2024), we hypothesize that children in the treatment group will exhibit greater gains in their EF skills from the fall to spring of preschool (see Fig. 1, for theory of change). Moreover, given the present study’s focus on publicly funded preschool programs in low-income school districts, the present study also allows us to understand the effects of PL for a diverse group of students, including children at risk for learning difficulties. Given that research suggests that teacher PL may be most beneficial for children learning in high-poverty school districts (e.g., Pianta et al., 2008), the present study has the potential to shed light on whether these benefits also extend to children’s EF development during preschool.

## 4. Method

### 4.1. Procedure

Our study team worked with teachers and administrators from participating preschool centers during the fall of the 2021-2022 school year to recruit families to participate in the present study. Recruitment occurred on a rolling basis, and parents/caregivers provided consent for their children to be remotely assessed, either at home or in school. Since COVID-19 protocols were in place at participating schools throughout the duration of the study, we conducted all child assessments via Zoom using a recently validated battery of remote EF measures (Ahmed et al., 2022). Children were tested in either their home or school, based on participant preference and/or availability. Home-based assessment sessions were proctored by caregivers, while school-based assessment sessions were proctored by substitute teachers, paraprofessionals, caregivers, and teacher aides. Families were either emailed or texted Qualtrics surveys consisting of demographic, home, and child-level questionnaires. Teachers were asked to answer questions about their educational background and certification, demographic information, as well as questions about program implementation.

### 4.2. Participants

Three hundred sixty children were recruited from 17 Head Start and Michigan’s Great Start Readiness Program centers. Of those, 152 children attended classrooms whose teachers received the intervention (*n* = 25), while 208 attended control classrooms (*n* = 24). Teachers in both treatment and control classrooms used HighScope’s preschool curriculum, which included enhanced PDR and CR curricular components; however, three treatment and four control classrooms used other curricula (i.e., The Creative Curriculum and Connect4Learning Curriculum). Given that the present investigation sought to isolate the treatment effects of PD and coaching on children’s EF skills, classrooms that did not use HighScope’s curriculum were excluded, resulting in an analytic sample of 317 children across 22 treatment (*n* = 141) and 20 control (*n* = 176) classrooms. Child-, family-, and teacher-level characteristics for the analytic sample are described below and reported separately by treatment and control conditions in Table 1. All procedures and protocols, including participant recruitment materials, were reviewed and approved by the Institutional Review Board at Michigan State University. Teachers and parents/caregivers of participating children provided written consent, and all children provided verbal assent before each testing session.

#### 4.2.1. Child and family characteristics

Children (Female = 55%), ranged from 35 to 63 months old (*M* = 49.8 months, *SD* = 6.4 months) when assessed in the fall. Of the parents/caregivers who completed the demographic survey (*n* = 250), approximately 38% reported their child’s race as White, 36.8% Black, 14.4% multiracial, 6.4% listed their race as ‘other,’ and 4.4% noted that they were Asian/Pacific Islander. In addition, 6% of parents/caregivers reported their children were Hispanic/Latine. Roughly 65% of mothers did not complete college (4.5% completed some high school, 30.9% graduated high school, and 29.6% completed some college), while 15.6% reported completing an undergraduate degree, and 19.3% completed graduate or professional school. All participating families reported that their child was proficient in English, which was the language in which all assessments were delivered. Approximately 31% of parents/caregivers reported that their child had mild medical or developmental concerns, ranging from language impairments to socioemotional and learning difficulties. However, no children were excluded from the present work due to an inability to participate for medical or developmental reasons.

#### 4.2.2. Teacher characteristics

Of the 42 teachers who participated in the study, 9.8% completed an associate’s degree, 56.1% completed a bachelor’s degree, and 34.1% completed a master’s degree. Moreover, 33.3% of all teachers were certified and completed either a ZA (Early Childhood Education: Pre-Kindergarten–Kindergarten) or ZS (Early Childhood Pre-Kindergarten: General and Special Education) endorsement. Finally, on average, teachers had 9 years (*SD* = 7.7 years) of prior teaching experience.

### 4.3. Randomization

Randomization occurred within schools at the classroom level, with classrooms being either assigned to treatment or control conditions, using a random number generator. For schools that had odd numbers of classes, one classroom was randomly chosen to be set aside into a separate pool before random selection of treatment and control classes; all classes in this separate pool were then assigned to a condition using a random number generator, with three being assigned to the treatment condition and two being assigned to the control condition.

### 4.4. Teacher training workshops

Lead and assistant teachers attended five training sessions from December through April of the school year. Each workshop was four hours in length. All training sessions followed a similar schedule. Each day began with an opening activity meant to build community and a sense of safety, comfort, and connection amongst participants. These opening activities were often adjusted to respond to teacher needs at the time, which were determined from coaching sessions. Teachers then engaged in reflection and application activities related to that month’s topic. Across training sessions, teachers were provided with opportunities to reflect on their takeaways from the training, brainstorm how the content relates to their classroom and student dynamics, and spend time developing an implementation plan and unique goals to begin using the learned strategies in their classroom setting. For later sessions, teachers were asked to reflect on how new content related to prior training. The following topics were presented in the order noted here: adult-child interactions, emotion awareness, problem-solving, conflict resolution, planning and recall, and work time focused on the importance of play.

### 4.5. Teacher coaching

Teaching teams were provided with coaching by an experienced educator to support the implementation of new strategies learned during the training sessions. Coaches (*n* = 9; 8 female) were experienced pre-k teachers or graduate students with prior teaching experience. Specifically, 8 coaches had an undergraduate degree and 1 coach completed a master’s degree in education, human development, or related fields. Coaches also had experience implementing the HighScope curriculum and had prior experience in early childhood consultation and teacher training. Coaches were trained by and received mentorship from a HighScope staff member who was involved in developing and implementing the teacher training workshops. The mentor coach has extensive experience in pre-k teaching, school administration, as well as teacher training and coaching. Coaches participated in ongoing meta-coaching sessions and were trained to use a customized version of cognitive coaching, which is a relationship-based framework that builds on teachers’ strengths through conversations structured around planning, reflection, and problem-solving (Ellison & Hayes, 2009). In addition to training and mentorship, the coaches and the mentor coach held meetings across the school year to address issues or shared experiences that enriched the coaching team.

Each teaching team participated in at least one coaching session after participating in each of the five training sessions. These sessions occurred remotely and lasted approximately one hour, on average. Before each coaching conversation, coaches observed teachers’ instruction for approximately one to two hours using video conferencing tools. Observations usually occurred earlier in the week to allow coaching sessions to take place on the same week as the observation. Most coaching sessions took place on Fridays and lasted one hour. Coaching was based on a relationship- and strengths-based model that encouraged self-directed learning. Teachers set their own goals related to the training content and were guided through reflection and planning conversations with their coach to reflect on their own practice. Coaches followed a conversation map specific to the topic they wanted teachers to consider. Each coaching session began with thought-provoking questions (e.g., “What felt successful? Why might that be?”), after which, coaches paused to allow teachers time to consider their responses. Coaches were encouraged to use nonverbal and verbal cues–including neutral expressions, paraphrasing, and questioning–to strengthen their relationship with teachers and promote teacher learning. All teaching teams participated in at least one coaching session per professional learning workshop (i.e., 5) and were provided with two additional sessions as needed or desired (Mean coaching sessions = 5.81; *SD* = 1.24).

#### 4.5.1. Fidelity of program implementation

All teachers in the treatment group completed all five training sessions. There were 10 occurrences when teachers could not attend a particular training session. In these instances, the teachers watched video recordings of the training sessions and completed the training activities on their own. There was full compliance in terms of coaching, with all teachers completing all 7 coaching sessions and attending all five training sessions.

### 4.6. Measures

Child assessments were conducted in school or at home in the fall and spring of the school year using Zoom Video Communications web conferencing technology. All participants were assessed using either a tablet or a computer. Each measure required varying levels of adaptation for remote administration (for complete details regarding remote task conversion and validation, see Ahmed et al., 2022). For example, measures that relied on visual stimuli were converted into Microsoft PowerPoint format. Tasks that required non-verbal responses from the child involved the assessor using Zoom’s remote-control function. Prior to each assessment session, parents/caregivers or proctors were asked to sit with the child during the assessment battery to interact with Zoom, move the tablet as needed, support the child’s engagement with the tasks, minimize distractions, and not provide children with prompts or answers to the assessment questions. A growing body of research suggests that remote EF assessments are a valid and reliable alternative to in-person assessment, with studies demonstrating their internal consistency, test-retest reliability, and predictive validity to various developmental outcomes (e.g., Ahmed et al., 2022; Lynch et al., 2023). Further, research comparing remote vs. in-person administration of child EF assessments has shown equivalence between the two approaches (Hamner, et al., 2022; Wright, 2020).

#### 4.6.1. Working memory

The Digit Span task (Wechsler, 1991) was administered to measure children’s working memory capacity, as the task requires storing, maintaining, and retrieving information held in short-term memory. In this task, children are asked to accurately recall and recite a string of numbers in the same order in which they were presented. The list of numbers increases by one item for each correct response recorded. If the participant responds incorrectly twice in a row, the instructor moves on to the next section of the task. The Digit Span task demonstrates excellent reliability and validity (Wechsler, 2008).

#### 4.6.2. Inhibitory control

The Day-Night Stroop task (Gerstadt et al., 1994) is a child-friendly adaptation of the original Stroop task. The Day-Night Stroop measures children’s inhibitory control by requiring children to say the word “day” when presented with a black card displaying a moon and stars and “night” when presented with a white card displaying a sun. The task includes two practice trials to ensure children understand the instructions, followed by 14 pre-randomized testing items: 7 requiring a “day” response and 7 requiring a “night” response. Children receive a score of 2 for each correct response, 1 for a self-corrected or similar response (e.g., “sun” instead of “day”), and 0 for an incorrect response. The Day-Night Stroop task has demonstrated good convergent validity (Carlson & Moses, 2001), internal reliability (von Stauffenberg & Campbell, 2007), and test-retest reliability (Thorell & Wahlstedt, 2006).

#### 4.6.3. Cognitive flexibility

The Dimensional Card Change Sort task (DCCS; Zelazo, 2006) requires children to sort a series of test cards (e.g., red rabbits and blue boats) by one dimension (e.g., shape) and then switch to sorting by another dimension (e.g., color). This task begins with two practice trial items, followed by six testing items for the shape game, and finally, six testing items for the color game. Children are presented each test card and instructed to place them into one of the two boxes that the assessor has designated for that card (e.g., “all the rabbits go here” or “all the red ones go here”). The DCCS task exhibits excellent test-retest reliability (Beck et al., 2011) and convergent and discriminant construct validity (Weintraub et al., 2013).

#### 4.6.4. Global executive function

The revised version of the Head-Toes-Knees-Shoulders task (HTKS-R; Gonzales et al., 2021) was used to assess children’s global executive function. In this task, children are prompted to perform a movement opposite from that indicated by the assessor (e.g., “touch your head” requires the child to touch their toes). In the second part of the task, children are taught another two prompts (e.g., shoulders and knees) and are required to perform both pairs of opposites together. Finally, the rules are reversed (e.g., head is paired with knees). Scoring for this task includes aggregating the practice and testing trials across all four blocks to yield a sum score. The HTKS-R task demonstrates excellent reliability and validity (Gonzales et al., 2021).

#### 4.6.5. Child, family, and classroom characteristics

Following What Works Clearinghouse standards regarding baseline equivalence (What Works What Works, 2017), we examined whether each of the child-, family-, and teacher-level characteristics presented in Table 1 differed significantly between the treatment and control conditions. Examination of baseline child- and family-level characteristics revealed standardized mean differences between intervention and control groups requiring statistical adjustment for the following variables: child age at baseline testing, testing location, sex, race/ethnicity, medical/developmental concern status, and mother’s educational attainment. At the teacher level, years of teaching experience, educational attainment, and certification/endorsement status differed significantly between the treatment and control conditions. Accordingly, each of these variables was included as a covariate in our model estimating the effect of program impact on children’s EF skills.

### 4.7. Analytic plan

Descriptive statistics were calculated using SPSS (version 29; IBM Corp, Armonk, NY, USA), while all substantive analyses were conducted in Mplus (version 8.6; Muthén & Muthén, 1998-2017). To account for missing data across variables, full information maximum likelihood (FIML) estimation was employed, allowing for the use of all available information. Additionally, the MLR estimator, which is robust to non-normal distributions, was used in all analyses (Asparouhov & Muthén, 2016). Standard errors were adjusted using the *Mplus* CLUSTER command to account for the nesting of children within classrooms.

#### 4.7.1. Missing data

The study sample had varying levels of unplanned missingness (ranging from 21.8% to 32.2%) across the study’s focal variables. At baseline, 248 children completed at least a portion of the EF battery, while 247 contributed EF data in the spring. Intervention status was unrelated to missingness. However, participants with missing data in the spring were more likely to be younger at the time of their fall assessment and were more likely to have mothers with lower educational attainment. Spring missingness was also related to the location in which children were tested during their baseline assessment (i.e., at home vs. in school), with children tested at school being more likely to have missing data in the spring. Although the inclusion of these variables as covariates was already justified based on the results of baseline equivalence testing, they also help to reduce potential bias due to missingness and adhere to missing-at-random assumptions (Graham, 2009).

#### 4.7.2. Factor analysis

Before testing program impact, we conducted a confirmatory factor analysis (CFA) of pre- and post-test EF measures to evaluate the latent EF structure and ensure that model fit was acceptable before using latent variables in our analyses. Based on a large body of evidence which suggests that EFs are a unidimensional construct in the preschool-aged children (see Nelson et al., 2016, for review), we tested a measurement model in which each fall and spring EF assessment loaded onto a single factor at each measurement occasion (i.e., fall and spring), with correlated residual variances between latent EF variables and corresponding indicators across waves. Model fit was assessed using the Comparative Fit Index (CFI), which compares the fit of a target model to the fit of an independent, or null, model, and the Tucker Lewis Index (TLI), with values between 0.90 and 1.00 indicating good model fit (Hu & Bentler, 1998). We also examined the Standardized Root Mean Square Residual (SRMR), which is the difference between the residuals of the sample covariance matrix and the hypothesized model, and the Root Mean Square Error of Approximation (RMSEA), with values less than .08 and .06, respectively, indicating good model fit (Hu & Bentler, 1999).

#### 4.7.3. Treatment effects

PL treatment effects on children’s EF skills were estimated using a regression framework in the *Mplus* program (version 8.6; Muthén & Muthén, 2017). In this model, a dichotomous variable representing treatment status (0 = control, 1 = treatment) was included as a predictor of children’s spring EF, while children’s baseline EF performance was controlled for. Additionally, standard errors were adjusted to account for the nested structure of the data (i.e., children nested within classrooms), and all child-, family-, and teacher-level variables that significantly differed by treatment status were included as covariates.

### 4.8. Transparency and openness

Materials and analysis code for this study are available by emailing the corresponding author. This study was not preregistered.

## 5. Results

**Fig. 2.**
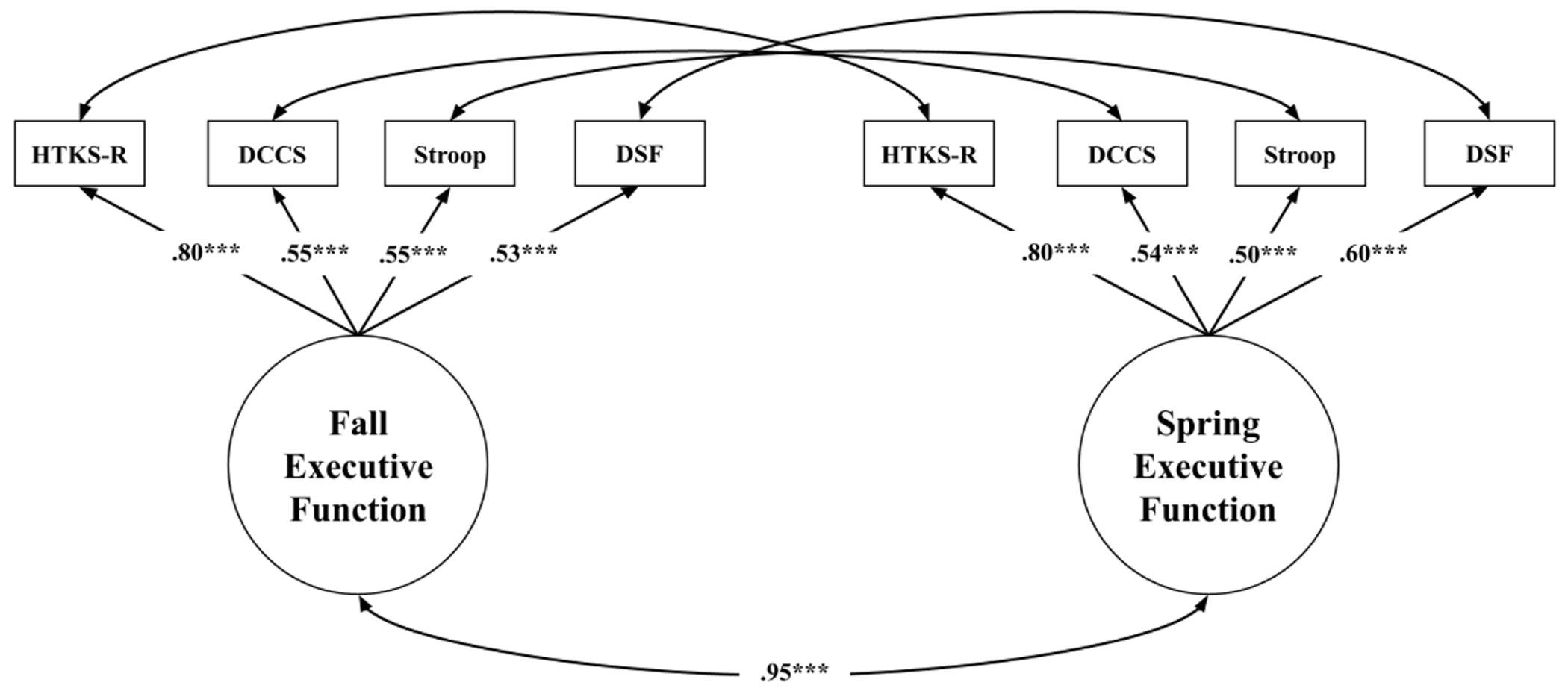
Confirmatory factor analysis of executive function measures. *Note:* Model fit statistics: χ^2^(15) = 20.130, *p* = .167, CFI = .991, TLI = .983; RMSEA = .034 (90% CI: .000, .068), SRMR = .031. *** p < .001. DCCS = Dimensional Card Change Sort Task; DSF = Digit Span Forward Task; HTKS-R = Revised Head-Toes-Knees-Shoulders Task; Stroop = Day/Night Stroop Task. Standard errors are adjusted to account for clustering within classrooms. The model was fit with MLR estimation, which provides maximum likelihood parameter estimates with standard errors and a mean-adjusted chi-square test statistic that is robust to non-normality. Residual variance estimates are omitted for presentation purposes.

**Fig. 3.**
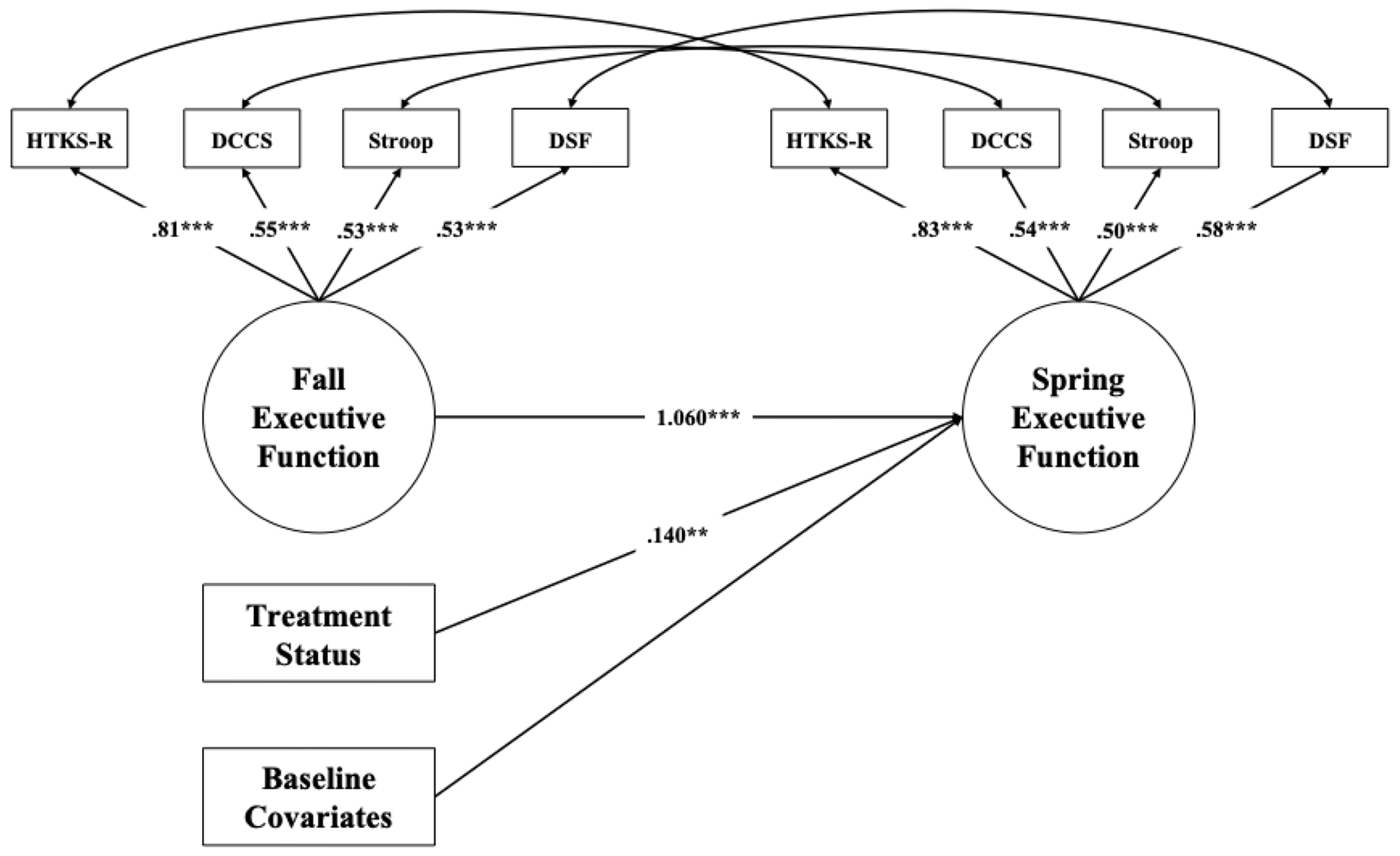
Impact of HighScope’s professional learning on children’s executive function. *Note:* Model fit statistics: χ^2^(81) = 106.78, *p* = .029, CFI = .963, TLI = .947; RMSEA = .032 (90% CI: .011, .047), SRMR = .040. ** p < .01, *** p < .001. DCCS = Dimensional Card Change Sort Task; DSF = Digit Span Forward Task; HTKS-R = Revised Head-Toes-Knees-Shoulders Task; Stroop = Day/Night Stroop Task. Standard errors are adjusted to account for clustering within classrooms. The model was fit with MLR estimation, which provides maximum likelihood parameter estimates with standard errors and a mean-adjusted chi-square test statistic that is robust to non-normality. Residual variance estimates are omitted for presentation purposes.

Descriptive statistics, reported by treatment and control group, are displayed in Table 1. Results from the factor analysis are presented Fig. 2 and the results from the multiple regression analyses are reported in Table 2 and displayed in Fig. 3.

### 5.1. Factor analysis

We fit the measurement model (i.e., CFA) using fall and spring assessments of the Digit Span, Day/Night Stroop, DCCS, and HTKS-R tasks, correlating the residual variances of each latent factor and corresponding indicators across waves. Standard errors were adjusted to account for nesting across classrooms. As is displayed in Fig. 2, the CFA provided an excellent fit to the data: χ^2^(15) = 20.130, *p* = .167, CFI = .991, TLI = .983, RMSEA = .034 (90% CI: .000, .068), SRMR = .031. CFI, TLI, RMSEA, and SRMR goodness-of-fit indices all exceeded the appropriate values for a determination of good model fit. Moreover, standardized factor loadings for all EF indicators were significant and exceeded a cutoff value of 0.40, ranging from 0.50 to 0.80 (Stevens, 2002). At the latent variable level, children’s fall and spring EF performance was positively correlated (*r* = .948, *p* < .001).

### 5.2. Treatment effects

After establishing the measurement model, we used multiple regression analyses in a latent variable framework to evaluate the impact of teacher PL on children’s EF development from fall to spring of the preschool year. In these analyses, child- and family-level covariates included children’s fall EF performance, age at baseline testing, testing location, sex, race/ethnicity, medical/developmental concern status, and mother’s educational attainment. At the teacher level, years of teaching experience, educational attainment, and certification/endorsement status were controlled for. The model demonstrated a good fit to the data: χ^2^(81) =106.781, *p* = .029, CFI = .963, TLI = .947, RMSEA = .032 (90% CI: .011, .047), SRMR = .040. Consistent with the CFA, all goodness-of-fit indices exceeding the appropriate values for acceptable model fit and standardized factor loadings remained significant and exceeded a cutoff value of 0.40. As reported in Table 2 and displayed in Fig. 3 over and above the contribution of child-, family-, and teacher-level covariates, treatment status positively predicted changes in children’s EF from fall to spring of preschool (*ß* = 0.140, S.E. = 0.060, *p* = 0.019).

### 5.3. Sensitivity analysis

We conducted a sensitivity analysis with the full sample (*N* = 360) to ensure our results were not affected by restricting the sample to only include classrooms that used HighScope’s curriculum. Program impact results using the unrestricted sample were interpretively unchanged (*ß* = 0.118, *S.E.* = 0.059, *p* = 0.046).

## 6. Discussion

As states across the country expand their public preschool programs, it is becoming increasingly important to identify the classroom practices that promote young children’s development. As such, interest in teacher professional learning (PL) programs has mounted in recent years. Despite this, experimental studies on the impact of PL on children’s outcomes remain limited, particularly as they relate to their executive function (EF) skills. Here, we implemented a randomized controlled trial to test the effect of HighScope’s PL on preschoolers’ EF development. HighScope’s PL model involves training and coaching by experienced educators to support teachers’ implementation of their preschool curriculum. Results from a sample of 317 preschool children enrolled in publicly funded preschools revealed a small, positive effect of teacher PL on children’s EF development during preschool. Overall, the results of the present study highlight the promise of PL for promoting children’s EF development during preschool.

### 6.1. Treatment effects of PL on EF development

Children exhibited significantly greater EF gains (effect size: *ß* = 0.140) when they attended classrooms where teachers received training and coaching. Although these gains were relatively small in terms of effect size, they point to the promise of teacher PL for supporting children’s EF development during preschool. Given ongoing debates about the efficacy of specialized pre-k curricula for promoting children’s EF (see Nesbitt & Farran, 2021, for review), it is important to consider the features associated with effective programming. All teachers in the present study used HighScope’s curriculum; however, only teachers in the treatment group received coaching and attended training workshops, suggesting that specialized curricula without additional support might not be effective in promoting children’s EF skills. This might explain some of the small and null effects of curriculum-based preschool interventions in the literature (Nesbitt & Farran, 2021).

It is possible that providing teachers access to PL to support the implementation of curricula is necessary for program efficacy. The present results support this notion; however, given the modest effect size, additional research is needed to better understand the PL content and dosage required to realize maximal gains in children’s EF. The PL model implemented in the present study included a total of five professional training sessions, each of which was four hours in length, plus one hour of coaching each month. It is possible that shorter and more frequent training sessions could be more effective, and there is some research that suggests this might translate to child outcomes during preschool. For example, Wasik and Hindman (2011) found that preschool teachers who attended shorter and more intensive training workshops and coaching sessions created higher quality classroom environments–leading to better student outcomes–compared to teachers who received Head Start’s business-as-usual PL.

Moreover, although several preschool interventions have reported null effects of curricular programs (e.g., Preschool PATHS, Incredible Years, Tools of the Mind), the present study implemented HighScope’s curriculum, which includes components designed to promote children’s EF and self-regulation. Specifically, Plan-Do-Review (PDR) and Conflict Resolution (CR) include instructional strategies designed to help children develop EF [e.g., sustaining attention (completing a task even when it is difficult); working memory (recalling and implementing work and play activities)], and self-regulation skills (e.g., encouraging children to reason, problem-solve, and resolve conflicts). Recent theoretical models suggest that curricula that focus on the cognitive and behavioral mechanisms that support EF development might be necessary to support children’s EF growth (see Mattera et al., 2021, for review). Importantly, research has suggested that PL that provides teachers with a theoretical understanding of the relevance of EF for children’s learning and development, as well as the classroom practices that promote children’s EF skills, might be an effective way to promote these skills during preschool (Muir et al., 2024). Results from a study by Muir and colleagues (2024) provided quasi-experimental evidence supporting these theoretical models–and the present randomized controlled trial provides additional, albeit modest, support for the effectiveness of programs that center professional learning as a mechanism of change for promoting preschooler’s EF development.

### 6.2. Implications for early childhood education

Although time spent in preschool has been associated with several beneficial outcomes for young children (Yoshikawa et al., 2013), large-scale studies have reported null and even negative effects of preschool attendance on social and behavioral development (Ansari et al., 2019; Bassok et al., 2018). Moreover, a recent study of the Tennessee Prekindergarten program reported negative long-term effects of preschool attendance for a host of developmental outcomes, an effect the authors hypothesize might be partially explained by a focus on academic content without regard to children’s socioemotional needs, including areas related to EF (Durkin et al., 2022). Similarly, using school cutoff methodologies, scholars have found that time spent in preschool benefited children’s academic development, but did not advance children’s EF relative to non-schooling factors (e.g., Skibbe et al., 2011). This suggests that business-as-usual preschool programs alone might not be effective in supporting all aspects of children’s development. Given that more than half of children living in the U.S. attend preschool (NCES, 2024), it is important to understand the features of preschool that can help meet all children’s needs. Here, we find promising results of teacher PL training and coaching for promoting children’s EF development. It is possible that training and coaching may serve as a starting point for recalibrating instructional emphases within preschool classrooms.

Moreover, it is also possible that providing teachers with instructional and behavioral support can foster teacher-child relationship quality. A large body of correlational research reports robust associations between teacher-child conflict and closeness and a host of developmental outcomes, including EF (e.g., Ansari et al., 2020; Berry, 2012). In addition to child outcomes, teacher training and coaching have been shown to reduce teacher stress, burnout, and increase workforce retention (Bassok et al., 2021; Roberts et al., 2020). The high rate of PL compliance and uptake in the present study, despite being implemented during a presumably high-stress COVID year, suggests that teachers themselves can also benefit from training workshops and coaching sessions. As states across the country continue expanding their public preschool programs, it is important to advance our understanding of the impact of early childhood education investments on teachers and students.

### 6.3. Limitations and future directions

Although there are various strengths of the present study, there are also limitations that are important to consider when interpreting the study’s findings. First, HighScope’s intervention was delivered to teachers and students during the COVID-19 pandemic, which has since been associated with detrimental effects on learning and development (e.g., Kuhfeld et al., 2022). There was also more likely to be heightened psychological stress among preschool teachers during the pandemic, which could have resulted in higher conflict in teacher-child relationships and heightened risk of professional turnover (e.g., Eadie et al., 2021). It is unclear how this might have affected the effectiveness of the intervention. On the one hand, it is possible that increased professional turnover during this time could have lessened treatment effects (Eadie et al., 2021). On the other hand, the relationship-centered approach used by the coaches employed by HighScope may have been particularly important for supporting the well-being of teachers during this time. Both of these COVID-19-related factors could have reduced EF growth across treatment and control groups and should be considered when interpreting the findings of the present study. Importantly, future research should examine whether these results generalize to typical preschool settings today, as the COVID-19 pandemic created unusual and potentially impactful confounds that may have affected the present study’s findings.

A second limitation of the current work is our lack of sufficient statistical power required to examine heterogeneous treatment effects across important demographic and child-level variables. Given that research has demonstrated that gaps in children’s EF development emerge prior to formal schooling (Ahmed et al., 2023; Morris et al., 2014), understanding for whom this intervention might be most effective could reveal important insights about program effectiveness. For example, Ansari and Pianta (2018) found that classroom age composition moderated the impact of a coaching intervention on preschoolers’ inhibitory control. Additionally, future research would benefit from examining variation in treatment effects across other important factors theorized to moderate early program effects, such as children’s baseline EF skills, sex, and socioeconomic status (Morris et al., 2014; Pianta et al., 2008; Weiland & Yoshikawa, 2013).

Finally, an important direction for future research is to understand the longitudinal effects of teacher PL on children’s developmental outcomes. The immediate gains in children’s EF at the end of preschool are certainly promising, but the longer-term impact, as children proceed through school, remains a critical and understudied area of research. Doing so has the potential to showcase the importance of preschool teacher PL, especially for under-resourced school districts like those represented in this study. Moreover, conducting longitudinal follow-up studies can provide a richer understanding of how PL programs might support children’s developmental outcomes across elementary school and beyond. This is especially important, as recent reviews have cast doubt on the benefits of preschool interventions for children’s long-term development (e.g., Burchinal et al., 2024).

## 7. Conclusion

Overall, the present study provides causal estimates of the impact of teacher PL on children’s EF skills while also contributing to the literature on preschool quality improvement and public preschool evaluations. Although modest, our findings showcase the potential of teacher PL for children’s EF development. Indeed, there are important advantages of specialized preschool curricula; however, it remains unclear how effective they are in promoting children’s EF development without supporting teachers’ implementation. Here, we found that pairing a curriculum with training workshops and coaching might be an effective way to promote preschoolers’ EF development. Nevertheless, more research is needed to understand the most effective dosage, for whom PL might be the most effective, and the extended effects on children’s developmental outcomes.

## Figures and Tables

**Table 1.** Descriptive statistics for full sample and treatment and control groups.

	Full sample	Treatment group	Control group
**Outcome variables in spring**			
Digit Span Forward Task	3.64 (0.91)	3.65 (0.84)	3.64 (0.97)
Day/Night Stroop Task	16.89 (9.32)	16.04 (9.10)	17.58 (9.46)
Dimensional Card Change Sort Task	8.69 (2.30)	8.63 (2.24)	8.74 (2.36)
Revised Head-Toes-Knees-Shoulders Task	42.37 (28.98)	41.89 (27.86)	42.76 (29.97)
**Baseline scores in fall**			
Digit Span Forward Task	3.26 (0.99)	3.28 (0.99)	3.25 (1.00)
Day/Night Stroop Task	11.77 (9.79)	9.94 (9.55)	13.27 (9.77)
Dimensional Card Change Sort Task	8.11 (2.52)	7.81 (2.37)	8.37 (2.62)
Revised Head-Toes-Knees-Shoulders Task	29.83 (24.24)	26.41 (19.68)	32.80 (27.34)
**Child and family characteristics**			
Age at baseline testing (in months)	49.83 (6.37)	48.44 (6.40)	51.02 (6.11)
Child tested in school	71.54%	73.33%	70.00%
Female	54.89%	59.57%	51.14%
Child race (Asian/Pacific Islander)	4.40%	9.17%	0.71%
Child race (Black/African American)	36.80%	33.03%	39.72%
Child race (White/Caucasian)	38.00%	38.53%	37.59%
Child race (Multiracial)	14.40%	12.84%	15.60%
Child race (Other)	6.40%	6.42%	6.38%
Child ethnicity (Hispanic/Latine)	6.05%	3.70%	7.86%
Mother’s education (some high school)	4.53%	1.91%	6.52%
Mother’s education (high school)	30.86%	29.52%	31.88%
Mother’s education (some college)	29.63%	28.57%	30.44%
Mother’s education (undergraduate degree)	15.64%	16.19%	15.22%
Mother’s education (graduate degree)	19.34%	23.81%	15.94%
Child has medical or developmental concern	30.65%	27.10%	33.33%
**Teacher characteristics**			
Years of teaching experience	9.01 (7.67)	6.61 (4.68)	11.65 (9.43)
Teacher education (associate’s degree)	9.76%	9.09%	10.53%
Teacher education (bachelor’s degree)	56.10%	54.55%	57.89%
Teacher education (master’s degree)	34.14%	36.36%	31.58%
Certified with ZA/ZS endorsement	33.33%	22.73%	45.00%

*Note*. Means (or percentages, where applicable) with standard deviations in parentheses. ZA = Early Childhood Education (Pre-Kindergarten-Kindergarten); ZS = Early Childhood Pre-Kindergarten - General and Special Education.

**Table 2.** Impact of HighScope’s professional learning on children’s executive function.

Predictor variable	Spring executive function		
	*β*	SE	CI
Treatment	.140**	.060	.023, .257
**Child and family characteristics**			
Baseline executive function	1.060***	.109	.846, 1.275
Age at baseline testing	−.105	.113	−.327, .116
Child tested in school	−.080	.063	−.203, .043
Female	.009	.067	−.122, .141
Mother’s educational attainment	−.076	.066	−.206, .054
Child medical/developmental concerns	.056	.055	−.052, .164
Race/ethnicity^1^			
Reference is: Other			
Black/African American	−.022	.075	−.169, .125
White/Caucasian	−.171	.093	−.353, .010
**Teacher characteristics**			
Years of teaching experience	.024	.046	−.067, .115
Educational attainment	−.012	.104	−.216, .193
Certified with endorsement	.063	.091	−.115, .240

Note. ß = standardized beta; CI = 95% confidence interval; SE = standard error.

1 Due to convergence issues, child race/ethnicity was recoded; “Other” includes Asian American/Pacific Islander, Hispanic/Latine, Multiracial, and Other.

* p < .05

** p < .01

*** p < 001.

## Data Availability

Data will be made available on request.
